# Supplementary material for: MukBEF-dependent chromosomal organization in widened Escherichia coli
Source: Front Microbiol. 2023 Mar 3;14:1107093. doi: 10.3389/fmicb.2023.1107093 (PMC10020239; doi:10.3389/fmicb.2023.1107093)
Supplement: Supplementary file 2 [file Image_1.pdf]

Supplementary Figures for

MukBEF-dependent chromosomal organization in widened *Escherichia coli*

Aleksandre Japaridze†, Raman van Wee†, Christos Gogou, Jacob W. J. Kerssemakers, Daan F. van den Berg and Cees Dekker*

† These authors have contributed equally to this work
***** Correspondence: Cees Dekker c.dekker@tudelft.nl
DOI: 10.3389/fmicb.2023.1107093

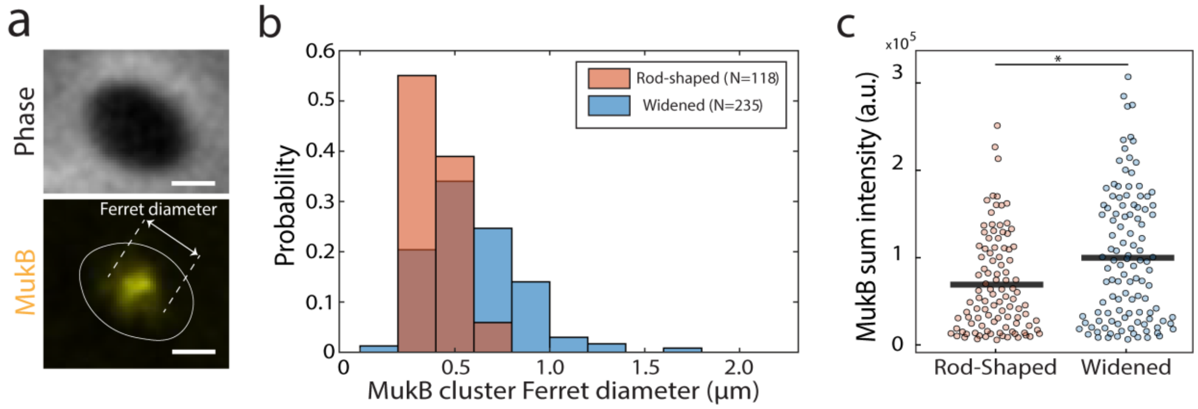

FIGURE S1

(A) Representative image of a widened *E. coli* cell in phase contrast (top) and the MukB-YFP fluorescence channel (bottom). Cell outline is indicated in white. Scale bar is 1µm and the Ferret diameter of the MukB cluster is indicated. **(B)** Ferret diameter distribution of MukB clusters in rod-shaped cells (red, *N=118*) and widened *E. coli* cells (blue, *N=235*). MukB clusters were significantly larger in widened cells compared to rod-shaped cells ($p < 0.0001$ with a single factor ANOVA test). **C**. MukB sum intensity profiles for rod-shaped cells (red, *N=101*) and widened *E. coli* cells (blue, *N=113*). The grey lines represent mean values. The mukB fluorescence signal in widened cells was slightly higher compared to rod-shaped cells (mean values 30% higher, $p = 0.015$ with a single factor ANOVA test).

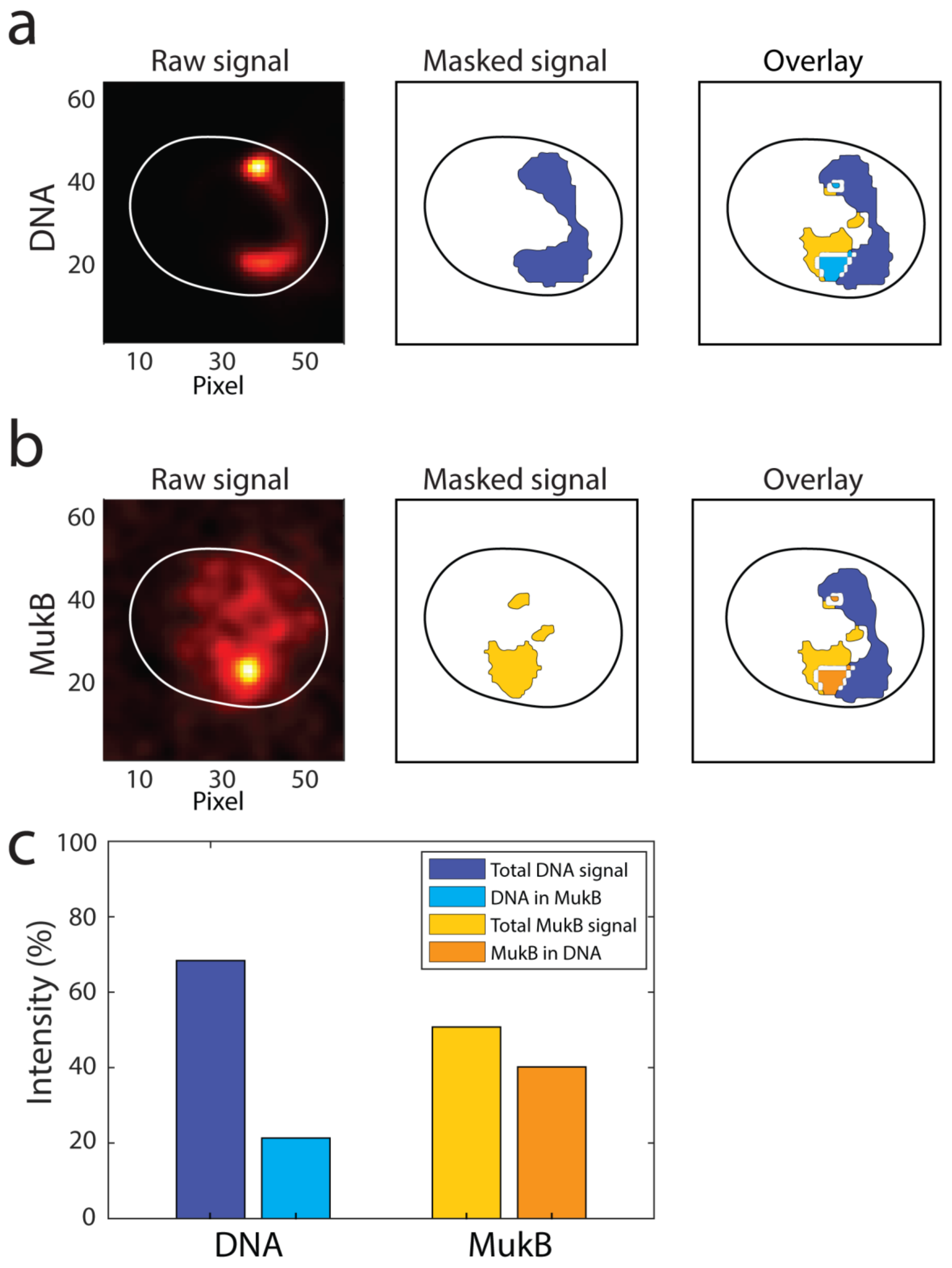

FIGURE S2

Quantitative co-localization of MukBEF signal with DNA signal. Fluorescent images of **(A)** DNA and **(B)** MukB channel. First the fluorescent signals are masked and then the masked signal of both channels is overlaid. Pixel size is 0.065 µm x 0.065 µm. Cell outline is indicated with white or black continuous lines. Note that the DNA intensity is color-coded using an exponential (and not linear) gradient and as a result, the low density ter region is too faint to be observed, although present. **(C)** The relative intensity overlap between the two channels (DNA and MukB) is calculated, by determining the fraction of total fluorescence intensity of one channel that lies within the masked region of the other channel.

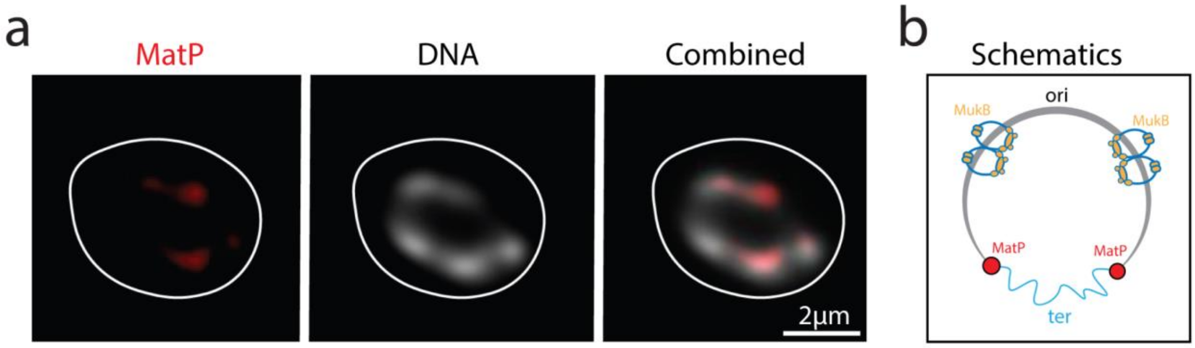

FIGURE S3

(A) Localization of MatP in widened cells. MatP (tagged with mCherry) (left), DNA (HU-mYpet) (middle) and an overlay of the two channels (right). Cell outline is indicated in white. The exact genomic location of the MatP spots is unknown, although, previous reports demonstrated that ter is organised as a low DNA density region (Wu & Japaridze 2019), i.e. on the right side of the cell shown here. In combination with earlier works showing that MatP binds exclusively inside the ter domain (Mercier et al., 2008), our images thus suggest that MatP binds at the edges of the ter region. **(B)** Schematics depicting the circular nucleoid of *E. coli* with the hypothetical position where MatP (red dots) binds and the flexible decondensed terminus region (blue wiggly line). MukBEF (dark blue and yellow) is positioned away from the terminus near the origin or replication.

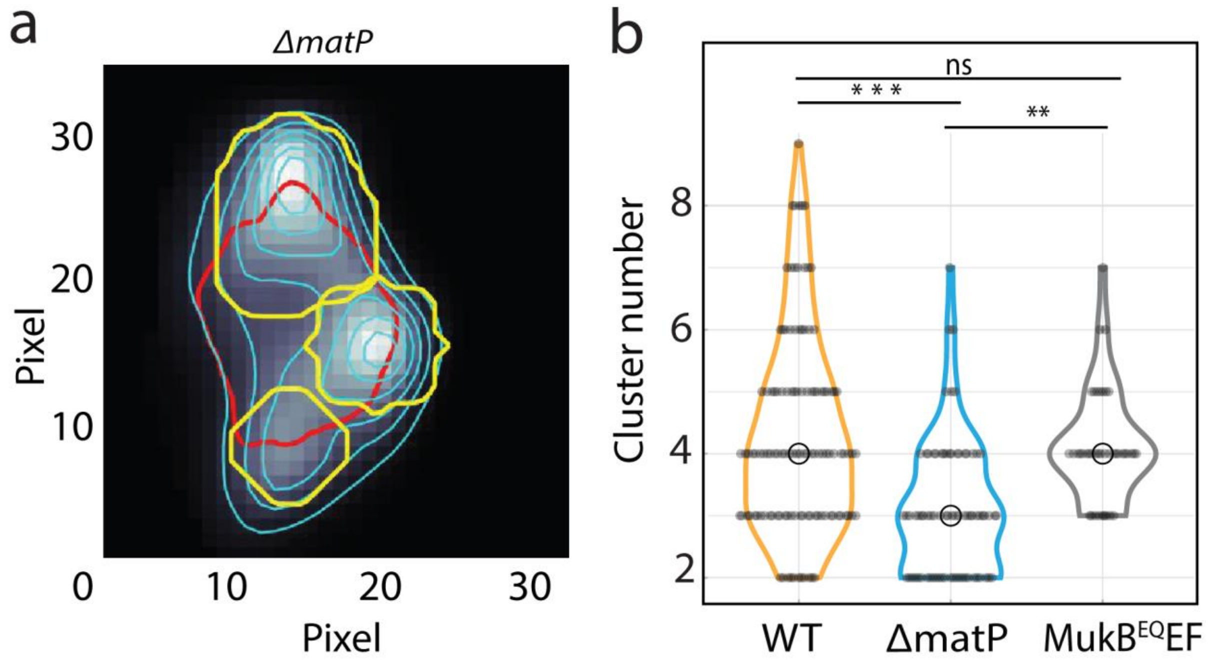

FIGURE S4

(A) Typical image of a chromosome of a *ΔmatP* cell after performing cluster analysis. The chromosome central ridge is shown in red line while the blue lines represent the intensity contour lines. Since the total chromosome DNA is constant, a smaller number of chromosome clusters indicates more compact chromosome. Yellow lines define DNA cluster contours. Pixel size is 0.065 µm x 0.065 µm. **(B)** Violin plot of the number of DNA clusters for various cell lines (wildtype in yellow, *ΔmatP* in blue, $MukB^{EQ}EF$ in grey). Black circles show the median values. Statistical significance was determined by performing a single factor ANOVA test. The following conventions are used: ns: $0.05 < p$, **: $0.001 < p < 0.01$, ***: $0.0001 < p < 0.001$.

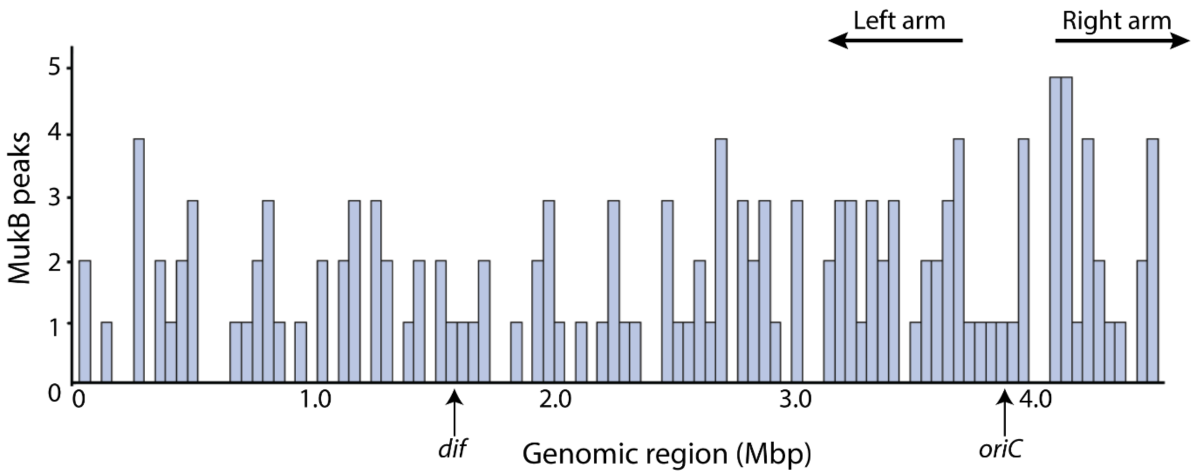

FIGURE S5

ChiP-Seq analysis of MukB binding along the *E. coli* genome. The genome occupancy of MukB protein, determined by ChIP-Seq (Nolivos et al., 2016), is plotted as a histogram (bin size 50 kbp) in which the bar height is indicative of relative binding enrichment. Peaks were identified by MACS v2.0.10.2013121616 (Zhang et al., 2008) using a maximum of 20 reads per unique position. Only highly enriched (greater than 2-fold enrichment over background) and highly significant (–log10 q value of greater than 30) peaks were used for the analysis, yielding 147 (MukB) significant peaks in the full genome. The figure was prepared in MACS using the processed ChIP-Seq data from Nolivos et al., 2016. There were 25 peaks in 0.5 Mb upstream of OriC and 20 peaks in 0.5 Mb downstream of OriC corresponding to a relative 20% difference.

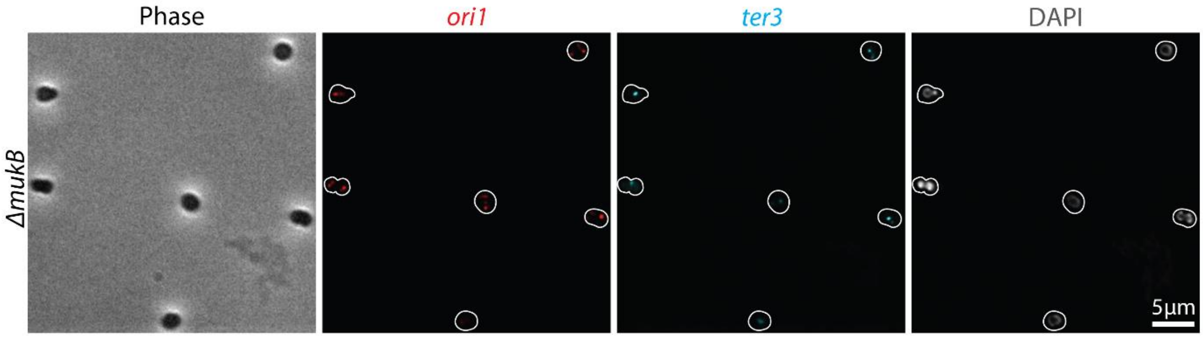

FIGURE S6

Microscopy images of widened *ΔmukB* cells. Phase contrast and the fluorescence channels of ori1, ter3 and DAPI (1 µg/ml) are shown. Cell outline is indicated in white.

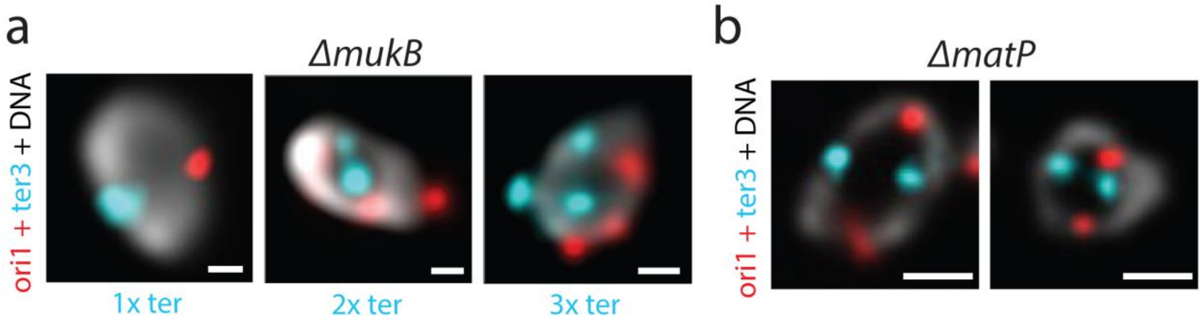

FIGURE S7

(A) Typical microscopy images of chromosomes in *ΔmukB* cells. Chromosomes display single, double or triple ori (red) and ter (cyan) foci. Scale bars are 1um. **(B)** Typical microscopy images of dimer chromosomes (with two ori (red) and two ter (cyan) foci) in *ΔmatP* cells.

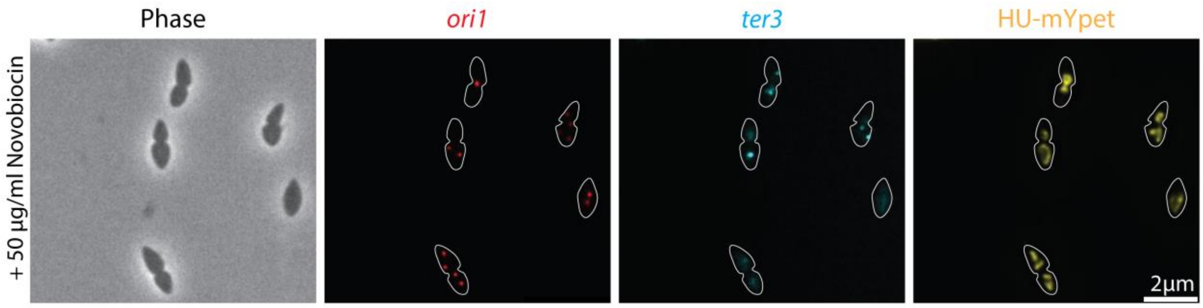

FIGURE S8

Microscopy images of replicating widened wildtype cells in the presence of Novobiocin (50µg/ml). Phase contrast and the fluorescence channels of ori1, ter3 and HU-mYpet are shown. Cell outline is indicated in white. Adding Novobiocin had a clear influence on the replication and segregation process in cells. Cells typically had misplaced ter loci with respect to the division septum and often had chromosomes placed in the middle of the cell, rather than at the cell poles. As a result, cells were unable to divide.

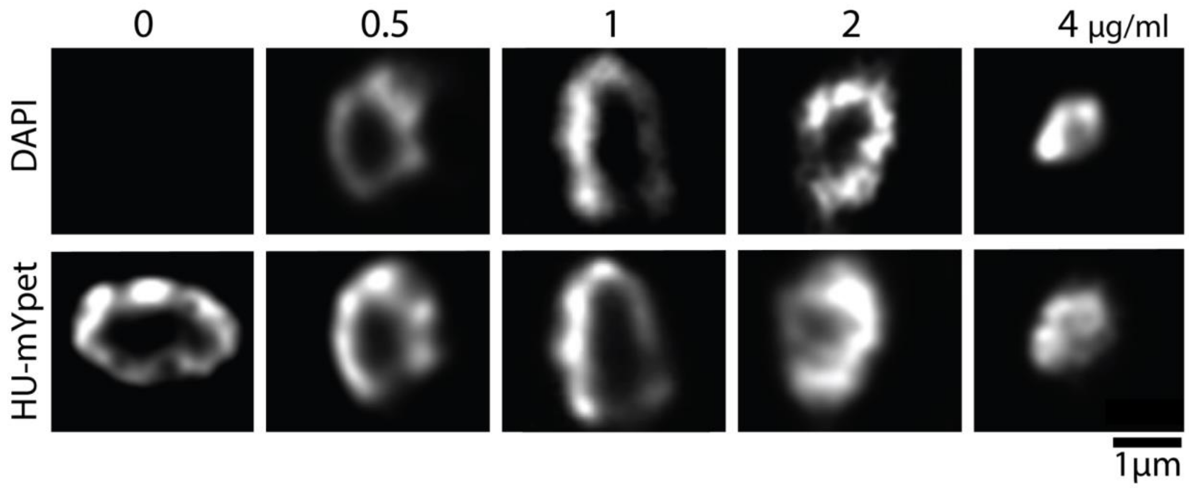

FIGURE S9

Microscopy images of circular chromosomes stained with various concentrations of DAPI and labelled with HU-mYpet. Top: The chromosome is stained with various concentrations of DAPI (0.5, 1, 2 and 4 µg/ml final concentration incubated for 1min). Bottom: The same chromosomes labelled with HUmYPet. High concentrations of DAPI alter the chromosome conformation and compact the nucleoid.

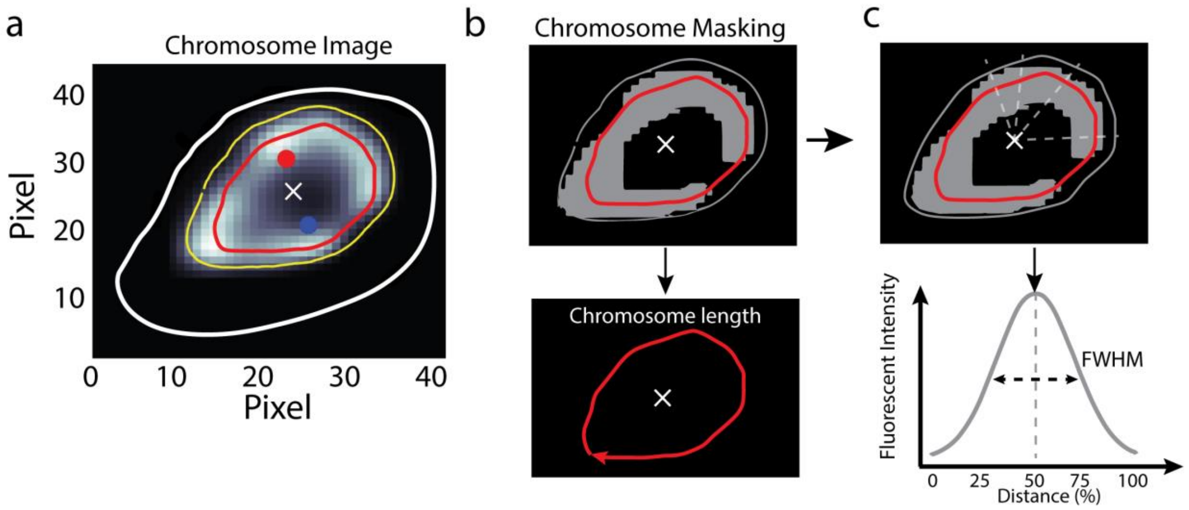

FIGURE S10

(A) Typical example of a circular chromosome. The red line depicts the 'backbone ridge', white line depicts cell contour, yellow line depicts the outer edge of the chromosome. Red and blue dots depict the position of the ori1 and ter3 foci. White cross depicts the chromosome centre of mass. Pixel size is 0.065 µm x 0.065 µm. **(B)** In order to identify the chromosome and extract geometric parameters, first the chromosome is masked (in grey). Chromosome length (reported in **Fig. 3B)** is defined as the length of the DNA backbone ridge (red line shown on panel a). **(C)** The width of the chromosome (reported in **Fig. 3C**) is defined as the average full-width-at-half-maximum (black dotted line) along the lines connecting the outer chromosome line and the chromosome centre of mass (dotted white lines) (*N=100* measurements per cell per 3.6° angle).

Supplementary References

Mercier, R., Petit, M. A., Schbath, S., Robin, S., El Karoui, M., Boccard, F., et al. (2008). The MatP/matS site-specific system organizes the terminus region of the *E. coli* chromosome into a macrodomain. *Cells* 135, 475–485. doi: 10.1016/j.cell.2008.08.031

Nolivos, S., Upton, A. L., Badrinarayanan, A., Müller, J., Zawadzka, K., Wiktor, J., et al. (2016). MatP regulates the coordinated action of topoisomerase IV and MukBEF in chromosome segregation. *Nat. Commun.* 7:10466. doi: 10.1038/ncomms10466

Wu, F., Japaridze, A., Zheng, X., Wiktor, J., Kerssemakers, J. W. J., and Dekker, C. (2019a). Direct imaging of the circular chromosome in a live bacterium. *Nat. Commun.* 10, 2194–2199. doi: 10.1038/s41467-019-10221-0

Zhang, Y., Liu, T., Meyer, C. A., Eeckhoute, J., Johnson, D. S., Bernstein, B. E., et al. (2008). Model-based analysis of ChIP-Seq (MACS). *Genome Biol.* 9:R137. doi: 10.1186/gb-2008-9-9-r137
